# Intravenous artesunate plus oral dihydroartemisinin–piperaquine or intravenous quinine plus oral quinine for optimum treatment of severe malaria: lesson learnt from a field hospital in Timika, Papua, Indonesia

**DOI:** 10.1186/s12936-019-3085-3

**Published:** 2019-12-30

**Authors:** Silvester Alexandro Sikora, Jeanne Rini Poespoprodjo, Enny Kenangalem, Daniel A. Lampah, Paulus Sugiarto, Ida Safitri Laksono, Riris Andono Ahmad, E. Elsa Herdiana Murhandarwati

**Affiliations:** 1 Postgraduate Programme of Tropical Medicine, Faculty of Medicine, Public Health and Nursing, Universitas Gadjah Mada, Yogyakarta, Indonesia; 2 Department of Paediatrics, Faculty of Medicine, Public Health and Nursing, Universitas Gadjah Mada, Yogyakarta, Indonesia; 3 Timika Malaria Research Programme, Papuan Health and Community Development Foundation, Timika, Papua Indonesia; 4 Mimika District Hospital, Timika, Papua Indonesia; 5 Mitra Masyarakat Hospital, Timika, Indonesia; 6 Center for Tropical Medicine, Faculty of Medicine, Public Health and Nursing, Universitas Gadjah Mada, Yogyakarta, Indonesia; 7 Dept. of Epidemiology, Biostatistics and Population Health, Faculty of Medicine, Public Health and Nursing, Universitas Gadjah Mada, Yogyakarta, Indonesia

**Keywords:** Severe malaria, Artesunate, Quinine, Dihydroartemisinin–piperaquine, Length of stay, Recurrence

## Abstract

**Background:**

Intravenous artesunate and its follow on full course dihydroartemisinin–piperaquine are the standard treatment for severe malaria in Indonesia. The current policy suggests that intravenous and oral quinine could be used when standard therapy is not available. Its pragmatic use of both treatment combinations in a field hospital is evaluated.

**Methods:**

A retrospective study among hospitalized malaria patients receiving intravenous anti-malarial treatments at Mitra Masyarakat Hospital, Timika from April 2004 to December 2013 was conducted. The length of hospital stay (LoS) and the risk of malaria recurrence within 28 days after hospital admission were compared between patients receiving intravenous artesunate and oral dihydroartemisinin–piperaquine (Iv Art + DHP) and those receiving intravenous and oral quinine (Iv + Oral Qu).

**Results:**

Of 10,514 patients requiring intravenous therapy, 2759 received Iv + Oral Qu and 7755 received Iv Art + DHP. *Plasmodium falciparum* infection accounted for 65.8% (6915), while *Plasmodium vivax*, Mixed infections, *Plasmodium malariae* and *Plasmodium ovale* were accounted for 17.0% (1789), 16.4% (1729), 0.8% (79) and 0.01% (2) of the infections, respectively. The majority of severe malaria hospital admissions were highland Papuans (78.0%, 8201/10,501). In total 49% (5158) of patients were older than 15 years and 3463 (32.9%) were children under 5 years old. The median LoS was shorter in patients receiving intravenous artesunate compared to those treated with intravenous quinine (median = 2 [IQR 1–3] versus 3 days [IQR 2–4], p < 0.0001). Patients treated with intravenous quinine had higher risk of being hospitalized longer than 2 days (aOR of 1.70 [95% CI 1.54–1.88], p < 0.0001). The risk of recurrences within 28 days after hospital admission was 1.94 times higher (95% CI aHR 1.57–2.39, p < 0.0001) in patients receiving intravenous quinine with follow on oral quinine treatment than in patients treated with DHP after intravenous artesunate therapy.

**Conclusions:**

Intravenous artesunate reduced the LoS of malaria patients and in combination with DHP reduced the risk of malaria recurrence within 28 days after hospital admission compared to those with Iv + Oral Qu treatment. Thus, ensuring continuous supply of intravenous artesunate and artemisinin-based combination therapy (ACT) should be a priority.

## Background

Malaria remains a global health problem. In 2017 the World Health Organization (WHO) estimates that there were about 219 million malaria cases in the world with 5% of the cases are in the South East Asia (SEA) Region [1]. Malaria associated mortality trends in SEA have halved from 39,800 deaths in 2010 to 19,700 in 2017 [1]. Globally *Plasmodium falciparum* and *Plasmodium vivax* infections are responsible for the greatest number of death among patients with severe malaria and this includes in Papua, Indonesia [1, 2]. Improvement of clinical management and treatment of severe malaria patient is required to prevent the risk of death, disability and the risk of recrudescent [3–5].

An effective severe malaria treatment should include both intravenous and oral anti-malarial drugs to achieve rapid clinical recovery and prevent recurrent parasitaemia [3]. Artesunate, an artemisinin derivative, is more effective for the treatment of severe malaria compared to intravenous quinine, resulting in a 23–35% lower risk of mortality in both Asia and Africa studies [6–8]. Intravenous artesunate is recommended by the WHO as the first-line treatment for severe malaria and should be followed with an effective oral artemisinin-based combination therapy (ACT) to prevent recrudescence [3, 7]. Despite the superior treatment profile of artesunate, intravenous quinine is still recommended as alternative therapy when artesunate or artemether are unavailable [3, 9]. Information on the real life effectiveness of severe malaria treatment that includes both intravenous anti-malarial therapy and its follow on oral treatment in malaria endemic area outside of Africa is currently lacking [4, 10, 11]. This study evaluates the use of intravenous quinine plus oral quinine (IV + Oral Qu) which was the first line treatment for severe malaria and its follow on oral treatment before treatment policy change in March 2006 and intravenous artesunate plus oral dihydroartemisinin–piperaquine (IV Art + DHP) after policy change at the local hospital in Timika (Papua-Indonesia). Although the anti-malarial drugs analysed were from different period of observation, this study provides insights on the effectiveness of current treatment recommendation in a field hospital.

## Methods

### Study site

Timika is located in the most eastern part of Indonesia (Papua Province) with the population about 200,000 during the study period [12]. The area is mostly forested with little variation in the climate [12]. The annual incidence of malaria was 876 per 1000 population in 2004 [13] and has declined to 450 per 1000 population in 2013 with *P. vivax* and *P. falciparum* are equally prevalent (Annual Health Report, Mimika District-2013). Between 2004 and 2006, 23% of patients admitted to hospital with malaria had severe disease, the majority of complications were severe anaemia, comma and respiratory distress syndrome either alone or in combination [2].

Until November 2008, Mitra Masyarakat Hospital (RSMM) was the only hospital in the region. Since December 2009 RSMM has received about 80% of patients presenting to hospital with malaria [14, 15]. RSMM has a functioning high care unit for critically ill patients and blood transfusion service is available 24 h 7 days.

### Study population

The ethnic groups in Timika are categorized into highland and lowland Papuans and non-Papuan Indonesians. The majority of occupation is associated to the local mining company [12]. Infectious diseases are still the predominant cause of morbidity and mortality in this region followed by chronic non-infectious diseases (Annual Health Report, Mimika District-2013; RSMM Hospital Statistics Report-2013).

### Study design

This was a retrospective study using secondary electronic data (a Q-Pro™ database) containing information on patient’s clinical and demographic details and clinical diagnosis made by the attending physician of each patient presentation between April 2004 and December 2013. The diagnosis was made according to the International Classification of Diseases 10 (ICD 10). The data were merged using patient’s unique identifier (Hospital Record Number) and date with electronic data from laboratory and pharmacy records.

At the RSMM, protocols dictate that all patients presenting with fever or history of fever or any patient with severe illness should be checked for malaria by microscopy using Giemsa stained thick blood smears. Thin blood smears were performed if the parasitaemia was too high to count by thick film examination. The hospital microscopists received refresher training annually.

Prior to 2006, the first-line treatment for severe malaria was intravenous quinine and continued with oral schizontocidal, which could be either oral quinine for 7 days, chloroquine alone for *P. vivax* malaria or chloroquine plus sulfadoxine–pyrimethamine for *P. falciparum* infections [16]. Treatment protocol at the hospital for severe malaria was revised to intravenous artesunate and DHP follow on oral treatment in March 2006 [17, 18].

### Hospital protocol for severe malaria management

During the study period, intravenous artesunate was given with a dose of 2.4 mg/kg body weight (BW) at 0, 12 and 24 h and then once daily. Anti-malarials were switched to DHP as soon as patient could tolerate oral treatment. Quinine infusion was given as a loading dose 20 mg/kg BW over 4 h and followed by 10 mg/kg BW infused over 4–8 h three times a day until oral quinine treatment starts.

Parasite count by microscopy was done daily in all patients admitted with severe malaria to review parasite clearance during hospitalization. Criteria of discharge of severe malaria patient were absence of fever and absence of severe signs and symptoms, able to tolerate oral treatment and no parasitaemia found in the blood smear.

### Definitions and outcome of interests

Severe malaria case is defined as hospital admissions with malaria and receiving intravenous anti-malarial drugs (artesunate or quinine). Local hospital protocols of severe malaria criteria and clinical management followed the WHO guidelines at the time of the study [7, 19]. The effectiveness of severe malaria treatment is defined as the length of hospitalization stay (LoS). Daily laboratory follow up was not available in the database and defining effectiveness was not possible with parasite clearance rate. An effective anti-malarial drug with rapid parasite clearance would result in early recovery and hospital discharge [7]. Since hospitalization days are affected by the presence of comorbidities, malaria hospital admissions with co-morbidity of tuberculosis, trauma, stroke and other non-malaria diagnosis were excluded from the analysis.

Recurrent malaria was defined as representation to hospital as either an inpatient or outpatient, with malaria within 28 days after initial hospitalization with malaria. Anthropometric measurements were only done routinely in children under 5 years old and nutritional status was assessed according to the WHO criteria for malnutrition during the study period [20]. Older children and adults would only be measured for their weight, height and arm circumference if they had physical signs of severe wasting. Severe anaemia was defined as a haemoglobin concentration less than 5 g/dl [7, 21].

### Statistical analysis

Data were analysed using SPSS vs 21.0 for windows software (IBM SPSS Statistics). Normally distributed data were compared by Student’s t-test. Data not conforming to a normal distribution were compared by the Mann–Whitney U test.

### Risk factors for prolonged LoS analysis

The Chi squared test with Yates’ correction or by Fisher’s exact test and odds ratios (OR) with 95% confidence intervals (CI) were used to compare all categorical risk factors for prolonged hospital stay more than 2 days. All significant risk factors with p value < 0.05 were entered to multiple logistic regression equation to analyse independent risk factors for prolonged hospital stay (adjusted OR).

### Risk factors for malaria recurrence analysis

Kaplan–Meier survival methods was used to analyse the risk of hospital representation with malaria within 28 days after hospital admission for each of the following variables: age group (0–< 1 year, 1–< 5 years, 5–< 15 years and ≥ 15 years), sex, pregnancy status, ethnic groups (non Papuan, lowland Papuan and highland Papuan), nutritional status (normal and severe malnutrition), *Plasmodium* species, anaemia (Hb < 5 g/dl) and intravenous-oral anti-malarial drug received (IvArt + DHP and Iv + Oral Qu). Cox proportional hazards regression models were used to identify risk factors for recurrent malaria. Univariate analysis to examine hazard ratios (HR) with 95% CI were performed for each variables and all variables were included in the multivariable models (adjusted HR).

In view of treatment policy change and the anti-malarial drugs were collinear with the year of observation, a subgroup analysis were performed to patients admitted after treatment policy change only.

### Ethical approval

The study was approved by the Medical and Health Research Ethics Committee (MHREC) Faculty of Medicine, Public Health and Nursing, Universitas Gadjah Mada, Yogyakarta, Indonesia (KE/FK/1228/EC/2018).

## Results

### Patient characteristics

**Fig. 1:**
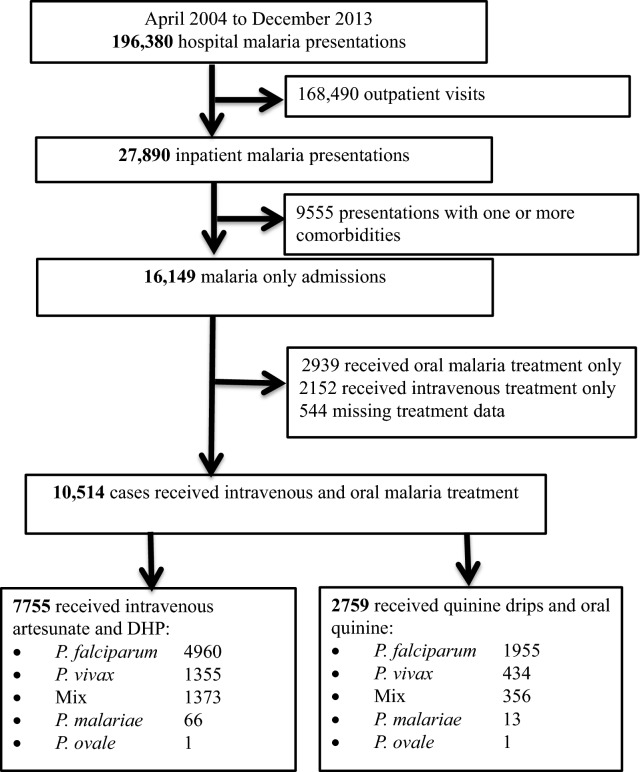
Study profile

From April 2004 to December 2013, a total of 27,890 patients were admitted to the hospital with a diagnosis of malaria and 34.2% (9555) of the cases had one or more co-morbidities. Of 16,149 malaria only admissions, 18.2% (2939) received oral malaria treatment only, 13.3% (2152) received intravenous anti-malarial only and 3.4% (544) had missing treatment data. This study focuses on the remaining 10,514 malaria patients receiving intravenous anti-malarial drugs and its follow on anti schizontocidal oral treatment of which 73.7% (7755) received IvArt + DHP and 2759 (26.3%) Iv + Oral Qu (see also Fig. 1).

**Fig. 2:**
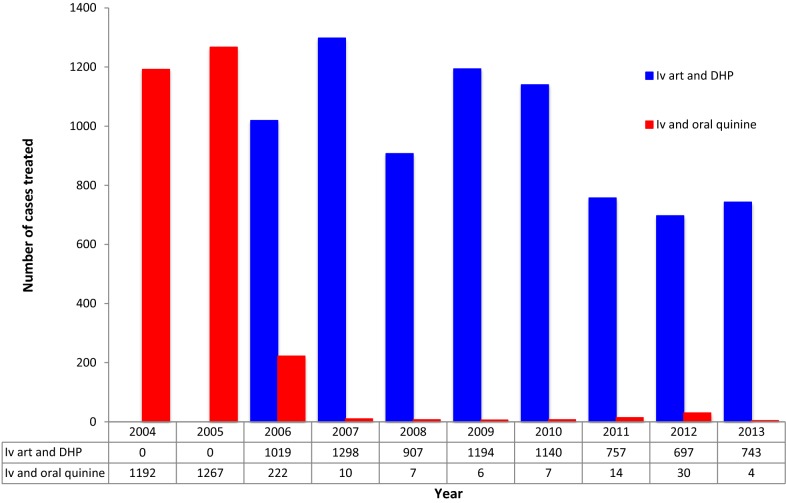
Treatment profile during the study period

Of those receiving both intravenous and oral anti-malarial treatment, *P. falciparum* accounted for 65.8% (6915), *P. vivax* 17.0% (1789), mixed infections 16.4% (1729), and *Plasmodium malariae* 0.8% (79) of the infections. There were two cases of *Plasmodium ovale*. Treatment prescriptions during the study period is presented in Fig. 2. Since treatment policy change in March 2006, intravenous artesunate has been the first-line treatment for severe malaria in the hospital and quinine were only prescribed in 83 patients, 59 (71.1%) of those were pregnant women.

The majority of patients were of highland Papuan ethnicity (78.0%, 8201/10,501), with the remainder being equally distributed between lowland Papuans (9%, 942) and non-Papuans (12.9%, 1358). Overall 5158 (49.1%) of patients were older than 15 years old with a median age of 14 (range 0.1–89) years. Infants and children under 5 years old accounted for 6.4% (677) and 26.5% (2786) of patients, respectively. Males accounted for 49.9% (5248) of patients. Four percent (405) of malaria admissions were pregnant women.

Severe malnutrition was recorded in 76 (0.7%) patients. Haemoglobin concentration (Hb) data was available in 90.6% (9530) patients of whom 18.6% (1771) were severely anaemic (Hb < 5 g/dl). The mean Hb concentration was 8.6 g/dl (95% CI 8.58–8.72). Baseline characteristics of patients stratified by treatment are presented in Table 1.

**Table 1:** Characteristics of severe malaria admissions during the study period

Characteristics	Malaria treatment	
	Intravenous artesunate and DHP (n = 7755)	Intravenous and oral quinine (n = 2759)
Age, median year (range)	12.9 (0.1–80.6)	16.0 (0.1–89.0)
Age group, n (%)		
0–< 1 years old	490 (6.3)	187 (6.8)
1–< 5 years old	2103 (27.1)	683 (24.8)
5–< 15 years old	1460 (18.8)	433 (15.7)
≥ 15 years old	3702 (47.7)	1456 (52.8)
Sex, n (%)		
Male	3871 (49.9)	1395 (50.6)
Pregnant women, n (%)	313 (4.0)	92 (3.3)

Ethnic group, n (%)	n = 7752	n = 2749
Highland Papuan	5968 (77.0)	2233 (81.2)
Lowland Papuan	682 (8.8)	260 (9.5)
Non Papuan	1102 (14.2)	256 (9.3)
Species of malaria, n (%)		
*P. falciparum*	4960 (64.0)	1955 (70.9)
*P. vivax*	1355 (17.5)	434 (15.7)
Mixed	1373 (17.7)	356 (12.9)
*P. malariae*	66 (0.9)	13 (0.5)
*P. ovale*	1 (0.0)	1 (0.0)
Nutritional status, n (%)		
Normal	7696 (99.2)	2742 (99.4)
Malnutrition	59 (0.8)	17 (0.6)

Haemoglobin	n = 7033	n = 2497
Hb, mean g/dl (95% CI)	8.9 (8.8–9.0)	7.9 (7.7–8.0)
Anaemia, n (%)		
Hb ≤ 5 g/dl	1180 (16.8)	591 (23.7)
Hb > 5 g/dl	5853 (83.2)	1906 (76.3)

### Hospitalization days

For LoS assessment, deaths were excluded from the analysis (n = 36). There were 34 deaths (0.4%) in IvArt + DHP group and 2 deaths (0.1%) in Iv + Oral Qu group (p = 0.004). All deaths among patients with quinine treatment occurred before treatment policy was changed into intravenous artesunate in March 2006. The median LoS of patients who died and receiving intravenous artesunate was 4 days (range 0–122 days) and in two patients died treated with intravenous quinine the time to death were 2 and 3 days following hospital admission.

**Fig. 3:**
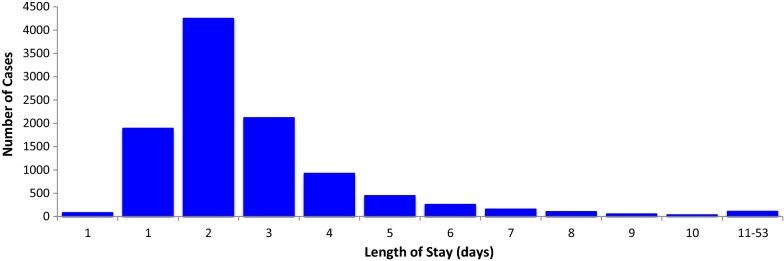
Length of stay and number of admissions

Among the 10,478 patients who were discharged, the median LoS was 2 days (interquartile range [IQR] 1–3 days; range 1–53). Most cases (99%, 10,365) were hospitalized ≤ 10 days and only 113 patients had a prolonged hospital stay (> 10 days) days. The profile of LoS is presented in Fig. 3.

The median LoS was shorter in the IvArt + DHP group compared to IV + Oral Qu group (median = 2 [IQR 1–3] versus 3 days [IQR 2–4], p < 0.0001). After controlling for other risk factors, the risk of hospitalizations more than 2 days were significantly increased in those receiving Iv + Oral Qu (aOR 1.70, 95% CI 1.54–1.88) compared to those treated with IvArt + DHP. After restricting the analysis to patients admitted after treatment policy change, IV + Oral Qu treatment remained as an independent risk factor for prolonged hospitalization (aOR 2.75, 95% CI 1.61–4.69), p < 0.0001. Being highland Papuans increased the risk of prolonged hospitalization by (aOR 1.19, 95% CI 1.04–1.36) compared to those of non-Papuans.

Malaria admission with severe malnutrition was more likely to have longer hospital stay (aOR 4.39, 95% CI 2.48–7.77) compared to those with normal nutritional status. Median LoS of malnourished patients with malaria were significantly higher (4 days [IQR 1–7]) than those of normal nutritional status (2 days [IQR 1–3], p < 0.0001). Having Hb < 5 g/dl increased the risk of prolonged hospitalization (aOR 3.8, 95% CI 3.10–3.78). Severe anaemia patients had longer LoS of 3 days (IQR 1–6) versus 2 days (IQR 1–3) in those with Hb ≥ 5 g/dl (p < 0.0001).

Pregnant women had higher risk of hospitalization more than 2 days (OR 2.0, 95% CI 1.73–2.71). Median LoS of pregnant women was 3 days (IQR 1–5) and those of non-pregnant individuals was 2 days (IQR 1–3, p < 0.0001). Children aged less than 15 years old appeared to have lower risk of extended stay. Compared to *P. falciparum* hospital admissions, other species of infections did not increase the risk of prolonged hospitalization (see also Table 2).

**Table 2:** Risk factors for longer hospital length of stay (> 2 days) (n = 10,478)

Risk factors	Hospital stay > 2 days, % (n/valid cases)	Univariable analysis		Multivariable analysis	
		OR (95% CI)	p	AOR (95% CI)	p
Age group					
≥ 15 years old	43.1 (2218/5144)	Reference		Reference	
0–< 1 year old	44.7 (302/675)	1.07 (0.91–1.26)	0.424	0.83 (0.69–0.99)	0.045
1–< 5 years old	37.5 (1041/2773)	0.79 (0.72–9.87)	< 0.0001	0.69 (0.61–0.78)	< 0.0001
5–< 15 years old	36.1 (680/1886)	0.74 (0.67–0.83)	< 0.0001	0.61 (0.54–0.68)	< 0.0001
Sex					
Male	39.7 (2082/5249)	Reference		–	–
Female	41.3 (2159/5229)	1.07 (0.99–1.16)	0.091	–	–
Pregnancy status					
Non pregnant	39.8 (4009/10,073)	Reference		Reference	
Pregnant	57.3 (232/405)	2.03 (1.66–2.48)	< 0.0001	2.17 (1.73–2.71)	< 0.0001
Ethnic groups					
Non Papuan	33.2 (449/1353)	Reference		Reference	
Lowland Papuan	34.8 (327/941)	1.07 (0.89–1.28)	0.435	1.01 (0.83–1.23)	0.904
Highland Papuan	42.3 (3458/8171)	1.48 (1.31–1.67)	< 0.0001	1.19 (1.04–1.36)	0.012
Nutritional status					
Normal	40.3 (4193/10,410)	Reference		Reference	
Malnutrition	70.6 (48/68)	3.56 (2.11–6.00)	< 0.0001	4.39 (2.48–7.77)	< 0.0001
Species of infections					
*P. falciparum*	41.2 (2840/6894)	Reference		–	–
*P. vivax*	37.3 (665/1782)	0.85 (0.76–0.95)	0.003	–	–
Mixed infections	40.8 (703/1722)	0.98 (0.88–1.10)	0.779	–	–
*P. malariae*	41.0 (32/78)	0.99 (0.63–1.56)	0.976	–	–
*P. ovale*	50 (1/2)	1.43 (0.09–22.83)	0.801	–	–
Anaemia (g/dl)					
Hb ≥ 5	36.1 (2797/7739)	Reference		Reference	
Hb < 5	66.5 (1169/1757)	3.51 (3.15–3.92)	< 0.0001	3.81 (3.39–4.28)	< 0.0001
Intravenous anti-malarials					
Intravenous artesunate and DHP	36.3 (2805/7721)	Reference		Reference	
Intravenous and oral quinine	52.1 (1436/2757)	1.91 (1.75–2.10)	< 0.0001	1.70 (1.54–1.88)	< 0.0001

### Risk of recurrence within 28 days after hospital admission

The risk of having malaria representations to the hospital within 28 days after admission was 6.1% (169/2759) in patients receiving Iv + Oral Qu and 3.2% (249/7755) in those treated with IvArt + DHP (aHR 1.94, 95% CI 1.57–2.39). After treatment policy change, the risk of recurrence within 28 days after hospitalization remained significantly greater in patients receiving Iv + Oral Qu (8.4%, 7/83) compared to those treated with IvArt + DHP (3.2%, 248/7729) with the adjusted HR of 4.91 (95% CI 2.02–11.89), p < 0.0001.

Infants and children under-fives had significant risk of having recurrence (aHR 3.97, 95% CI 2.81–5.59; 2.99, 95% CI 2.32–3.86) respectively compared to the 15 years older age group. Being highland Papuan also increased the risk of malaria recurrence (aHR 2.70, 95% CI 1.57–4.66) compared to non-Papuans. No recurrence was found in patients with severe malnutrition. Species at initial admissions and being pregnant did not increase the risk of malaria recurrence (see also Table 3).

**Table 3:** Risk for recurrence within 28 days after hospital discharge

Risk factors	Recurrence within 28 days, % (n of events/total cases)	Univariable analysis		Multivariable analysis	
		HR (95% CI)	p	AHR (95% CI)	p
Age group					
≥ 15 years old	2.3 (118/5158)	Reference		Reference	
0–< 1 year old	9.6 (65/677)	4.31 (3.19–5.84)	< 0.0001	3.97 (2.81–5.59)	< 0.0001
1–< 5 years old	6.6 (184/2786)	2.96 (2.35–3.73)	< 0.0001	2.99 (2.32–3.86)	< 0.0001
5–< 15 years old	2.7 (51/1893)	1.18 (0.95–1.65)	0.311	1.18 (0.83–1.68)	0.364
Sex					
Male	3.6 (188/5266)	Reference		Reference	
Female	4.4 (230/5248)	1.23 (1.02–1.49)	0.034	1.21 (0.98–1.48)	0.073
Pregnancy status					
Non pregnant	4.0 (407/10,109)	Reference		Reference	
Pregnant	2.7 (11/405)	0.68 (0.37–1.23)	0.200	0.88 (0.46–1.70)	0.705
Ethnic groups					
Non Papuan	1.2 (16/1358)	Reference		Reference	
Lowland Papuan	2.9 (27/942)	2.44 (1.31–4.53)	0.005	1.64 (0.85–3.16)	0.142
Highland Papuan	4.6 (375/8201)	3.96 (2.39–6.52)	< 0.0001	2.70 (1.57–4.66)	< 0.0001
Nutritional status					
Malnutrition	0.0 (0/76)	Reference		Reference	
Normal	4.0 (418/10,438)	20.28 (0.10–3994.69.25)	0.169	–	–
Species of Infections					
*P. falciparum*	3.6 (247/6915)	Reference		Reference	
*P. vivax*	4.9 (88/1789)	1.39 (1.09–1.77)	0.007	1.11 (0.85–1.46)	0.446
Mixed infections	4.7 (81/1729)	1.32 (1.03–1.69)	0.029	1.16 (0.88–1.52)	0.301
*P. malariae*	2.5 (2/79)	0.71 (0.18–2.86)	0.633	0.92 (0.23–3.71)	0.907
*P. ovale*	0 (0/2)	–	–	–	–
Anaemia (g/dl)					
Hb ≥ 5	3.6 (283/7759)	Reference		Reference	
Hb < 5	5.1 (90/1771)	1.42 (1.12–1.79)	0.094	0.90 (0.70–1.15)	0.403
Intravenous anti-malarials					
Intravenous artesunate and DHP	3.2 (249/7755)	Reference		Reference	
Intravenous and oral quinine	6.1 (169/2759)	1.91 (1.57–2.33)	< 0.0001	1.94 (1.57–2.39)	< 0.0001

## Discussions

Intravenous artesunate is highly effective for severe malaria treatment and has been widely used in malaria endemic areas in Africa, America and the Asia–Pacific regions [3] The WHO recommends its use to any species of infections (*P. falciparum* and *P. vivax*) with severe manifestations [7, 21]. Ensuring sustainability of artesunate and ACT supplies remains a major challenge in most malaria endemic areas and in view of this, quinine is recommended as an alternative therapy [22].

The study findings highlight real life effectiveness of intravenous artesunate and intravenous quinine prescribed in a field hospital to patients with severe malaria. It was found that patients receiving intravenous artesunate had significantly shorter hospitalization days compared to those treated with intravenous quinine (median 2 versus 3 days). In addition, the risk of hospitalization longer than 2 days was 1.7 times higher in patients receiving intravenous quinine compared to those treated with artesunate. Consistent finding was also observed in subgroup analysis restricted to the period after treatment policy change suggesting minimal effect of possible shift in treatment practice to duration of stay. Artesunate is known for its rapid parasite clearance time compared to those of quinine [23, 24] and its use is associated with more rapid clinical improvement and early discharge from the hospital [25]. Similar shorter time to discharge was also described in an observational implementation study comparing intravenous artesunate and intravenous quinine in Congo (median 2 versus 3 days) [11].

However, the association of LoS and the choice of intravenous anti-malarial drugs is not straightforward. Rapid parasite clearance in patients admitted already in an advance state of severe complications would not improve the overall patient’s condition as further supportive therapy is still required to manage complications. The LoS in a controlled study in which strict severe criteria for admission was applied, intravenous artesunate had similar duration of hospitalization compared with quinine, which were 5 to 6 days [10, 23]. Intravenous artesunate has been shown to reduce the risk of mortality and therefore there is a potential to increase LoS [10].

The threshold of severe criteria assessment in patients with parasitaemia in a hospital setting like in Timika, is likely to be lower than that in a controlled study. This could explain the greater reduction of LoS in patients receiving a highly effective treatment found in this study. Previous observational study in this region has shown that the majority of severe complications at the same hospital were severe anaemia, followed with respiratory distress and impaired of consciousness [2].

Another significant risk factor for prolonged hospitalization was severe anaemia (Hb < 5 g/dl). Longer hospital stay in severely anaemic patients is very likely due to supportive treatment, which is multiple blood transfusions rather than delayed parasite clearance. Severely malnourished patients were more likely to stay longer in the hospital (aOR = 4.39) compared to those with normal nutritional status. Fluid, electrolyte and nutrient imbalance in patients with malnutrition are more likely associated with longer hospital days.

Being highland Papuan also increased the risk of prolonged hospitalization compared to non Papuans. Both ethnic groups are known to be more susceptible to malaria compared to those of the lowland Papuans [13]. Malaria treatment efficacy is also determined by the level of acquired immunity and risk of exposures to malaria [24]. Despite similarly being vulnerable to malaria, non Papuans are more likely to seek early medical attention for their illness and thus early recovery. Children younger than 15 years old had earlier time to discharge from the hospital compared to older individuals, which is consistent with the current knowledge that the duration of illness in children with severe malaria is shorter than in adults [7]. The longer hospital stay found in pregnant women with malaria in this study to a greater extent could be explained by concomitant obstetric conditions found during hospitalization. Unfortunately, details on obstetric diagnosis could not be obtained from the database.

A follow on oral anti-malarial agents after intravenous treatment should be given in patients with severe malaria to achieve optimum parasite clearance and prevent recrudescence [7]. This study showed that intravenous artesunate followed with unsupervised DHP for 3 days had lower risk of representations with malaria within 28 days (3%) after hospital admission compared to those receiving unsupervised oral 7-day quinine after intravenous quinine (6%) with almost 2 times higher risk of recurrence found in IV + Oral Qu group (aHR = 1.94).

DHP, an artemisinin-based combination therapy, for 3 days is highly efficacious for both falciparum and vivax malaria [26]. Piperaquine as the partner drug which has a long elimination half-life (≈ 28 days) will clear the remaining parasites and could also provide post treatment prophylactic effect [27, 28]. The 28 days cure rate of supervised 7 days quinine in multidrug resistant malaria area in Thailand was 87% and in Sudan was 93.7% [29, 30]. The main challenge is ensuring compliance of 3 times a day for 7 days quinine in a non-research environment [3, 22]. In addition several adverse effects of quinine, such as tinnitus, headache, nausea and dizziness is likely to reduce treatment adherence [3, 22]. Unsupervised 7 days quinine either alone or in combination with doxycycline in this region had a high recurrence rate at day 28 of 67% [16]. This explains the higher risk of recurrence in patients with oral quinine follow on treatment found in this study. It has been suggested that a follow on with ACT in patients receiving intravenous quinine is preferable [4].

Infant and young children were also at higher risk of having recurrence with the aOR of 3.97 and 2.99 respectively compared to those aged more than 15 years old. This group has been known to have less immunity and more vulnerable to malaria and recurrence episodes of malaria [31–33]. Compared to non-Papuan, highland Papuans were more likely to have recurrence (aHR = 2.70). This could be due to non-Papuans are more likely adhering to treatments which to some extent could be associated with higher socio-economic status found in this ethnic groups (Timika household data 2013, unpublished).

Interestingly, none of patients with severe anaemia had recurrences. It has been shown that iron deficiency is protective to infections, including malaria [34]. It is plausible that severely malnourished patient are iron deficient and thus relatively protected from recurrent malaria [35].

Intravenous artesunate has been proven to significantly reduce the risk of mortality compared to quinine in multicenter controlled intervention studies [6, 8]. This observational study found that mortality risk was greater following intravenous artesunate (0.4%) compared to those receiving intravenous quinine (0.1%). However, in view of treatment policy change in March 2006, the result should be interpreted with caution, as anti-malarial agents analysed in this study is collinear with the year of observation that leads to major changes in treatment seeking and admission practice. A 9 year evaluation of malaria morbidity and mortality trends before and after treatment policy change in March 2006 in the same hospital showed that malaria attributable mortality risk drops significantly from 0.53 to 0.32% and after DHP is widely used for the treatment for uncomplicated malaria, the number of malaria requiring hospital admission fell significantly from 14 to 7%, suggesting that after March 2006 patients admitted to the hospital and received intravenous artesunate may have been sicker than those admitted before policy change [15]. The longer median time to deaths in patients receiving intravenous artesunate (4 days, range 0–122 days) compared to those treated with intravenous quinine (2 and 3 days) may explain that artesunate delayed deaths in patient admitted with later stage of severe malaria who would otherwise have died earlier. Although the degree of clinical and laboratory severity could not be ascertained in this study, the longer time of deaths associated with the degree of severity in patients receiving intravenous artesunate were also found in a large multicenter randomized controlled trial comparing intravenous artesunate and quinine [10].

This study has several important limitations. Firstly, due to the nature of this study, parasite clearance time (which is an indicator for treatment effectiveness) could not be assessed. LoS is used as a proxy indicator of effectiveness with the assumption that parasite clearance will improve clinical outcome and shorter duration of stay [25].

Secondly, malaria recurrences in the population were not actively detected. This study only includes hospital malaria representations to examine recurrence. It is possible that some recurrence occur in the community or presents to other health facilities. However, RSMM provides free medical care for the local tribes and about 80% of malaria presentations in the district was to RSMM.

Lastly, this study is not designed for clinical trial. The type and degree of severe manifestations could not be ascertained in this study. However, the hospital protocol suggests that intravenous treatment should only be given to malaria with severe complications and to some extent could be used as an indicator of presence of severity. In about 10% of the cases, intravenous treatment were given to those unable to receive oral treatment due to comorbidity with other illnesses such as surgical, neurological and metabolic diseases cases. Therefore, malaria with significant co-morbidity were excluded from the analysis.

## Conclusion

The ultimate goal of severe malaria treatment is to clear parasites rapidly and prevent death, provide standard management of complications and prevent recrudescence [7]. This study highlights that in real life settings, intravenous artesunate followed with 3 days DHP is more effective in reducing the LoS (as proxy indicator of effectiveness) and recurrence within 28 days after hospital admission compared to intravenous quinine followed with 7 days oral quinine. Ensuring continuous supply of intravenous artesunate and oral ACT should be a priority.

## Data Availability

The datasets used and/or analysed during the current study are available from the corresponding author on reasonable request.

## Abbreviations

SEA: South East Asia
ACT: artemisinin-based combination therapy
Iv Art + DHP: intravenous artesunate plus dihydroartemisinin–piperaquine
Iv + Oral Qu: intravenous and oral quinine
LoS: length of stay
RSMM: Rumah Sakit Mitra Masyarakat
Hb: haemoglobin
aOR: adjusted odds ratio
aHR: adjusted hazard ratio
